# Hypotension with and Without Hypertensive Episodes During Endoscopic Adrenalectomy for Pheochromocytoma or Paraganglioma—Should Perioperative Treatment Be Individualized?

**DOI:** 10.3390/jcm13237054

**Published:** 2024-11-22

**Authors:** Akos Tiboldi, Jonas Gernhold, Christian Scheuba, Philipp Riss, Wolfgang Raber, Barbara Kabon, Bruno Niederle, Martin B. Niederle

**Affiliations:** 1 Department of Anaesthesia, Intensive Care Medicine and Pain Medicine, Division of General Anaesthesia and Intensive Care Medicine, Medical University of Vienna, 1090 Vienna, Austria; akos.tiboldi@meduniwien.ac.at (A.T.); jonas.gernhold@gmail.com (J.G.); barbara.kabon@meduniwien.ac.at (B.K.); 2 Division of Visceral Surgery, Department of General Surgery, Medical University of Vienna, 1090 Vienna, Austria; christian.scheuba@meduniwien.ac.at (C.S.); philipp.riss@meduniwien.ac.at (P.R.); bruno.niederle@meduniwien.ac.at (B.N.); 3 Division of Endocrinology and Metabolism, Department of Medicine III, Medical University of Vienna, 1090 Vienna, Austria; wolfgang.raber@meduniwien.ac.at

**Keywords:** pheochromocytoma, hypotension, alpha-receptor blocker, adrenalectomy, risk factor

## Abstract

**Background**: Hemodynamic instability is common during adrenalectomy for pheochromocytoma and paraganglioma (PPGL). Most analyses focus on the risk factors for intraoperative hypertension, but hypotension is a frequent and undesirable phenomenon during PPGL surgery. This study aimed to analyze the risk factors for hypotensive episodes during the removal of PPGL, and whether these episodes are always associated with concomitant intraoperative hypertensive events. **Methods**: A consecutive series of 121 patients (91.7% receiving preoperative alpha-blockade) treated with transperitoneal endoscopic adrenalectomy at a university hospital were analyzed, and pre- and intraoperative risk factors for intraoperative hypotension with or without intraoperative hypertension were analyzed using univariable and multivariable logistic regression analyses. **Results**: In total, 58 (56.2%) patients presented with intraoperative hypotension. Of these, 25 (20.7%) patients showed only hypotensive episodes but no hypertensive episodes (group 1), and 43 (35.5%) patients had both intraoperative hypotension and hypertension (group 2). The remaining 53 patients did not present with hypotension at all (group 3). When comparing group 1 (hypotension only) to all other patients with incidental diagnosis, higher age and lower preoperative diastolic arterial blood pressure (ABP) were significant risk factors for intraoperative hypotension; only the latter two were still significant in multivariate analysis. The significant risk factors for hypotension independent of hypertension (group 1 + 2 vs. group 3) were age and incidental diagnosis, pre-existing diabetes mellitus, and intraoperative use of remifentanil. Incidental diagnosis and use of remifentanil reached the level of significance in multivariate analysis. **Conclusions**: Since older age, incidental diagnosis of PPGL, lower preoperative ABP, and diabetes mellitus are risk factors for intraoperative hypotension, preoperative alpha-blocker treatment should be individualized for those at risk for hypotension. In addition, remifentanil should be used cautiously in the risk group.

## 1. Introduction

Pheochromocytoma and abdominal paraganglioma (PPGL) are adrenal (pheochromocytoma) or extra-adrenal (paraganglioma) tumors derived from neuroendocrine chromaffin cells that produce and secrete catecholamines (noradrenalin, adrenalin, and dopamine) and their metabolites ([nor]-metanephrines and 3-methoxytyramine). Whereas metanephrines are biologically inactive and are released continuously into the bloodstream [1], the release of catecholamines from vesicles is often episodic and [2] can cause the “classical” intermittent symptoms of (severe) hypertension or tachycardia associated with headache and diaphoresis. Recent studies indicate that most PPGL cases are nowadays detected incidentally as adrenal masses during abdominal imaging (ultrasound, CT, MRI) for reasons unrelated to adrenal tumors (adrenal incidentalomas) [3]. Additionally, patients with hereditary tumor syndromes (von Hippel–Lindau disease [VHL], Neurofibromatosis Type 1 [NF1], Multiple Endocrine Neoplasia Type 2 [MEN2], Succinate Dehydrogenase mutations [SDHx]) are routinely screened for PPGL, leading to earlier detection in these families, often with smaller tumor sizes [4]. Diagnosis is confirmed by elevated catecholamines in urine or more sensitively by free metanephrines in plasma or urine [5,6,7]. The prevalence of hypertension in patients with PPGL was found to be more than 80% [8], but evidence exists that hypertension is not specific to PPGL, as it is equally prevalent in control groups without PPGL [8,9]. Paroxysmal hypertensive episodes (hypertensive crisis) can be caused by sudden catecholamine release indirectly induced by emotional or physical exertion or directly by physical tumor manipulation during exercise and intraoperatively during tumor mobilization and resection [10]. Preoperative alpha-adrenoreceptor blockade (in addition to beta-adrenoreceptor blockade) has been the recommended premedication for patients with PPGL planned for surgery with the rationale of preventing complications due to catecholamine excess (myocardial infarction, cardiac insufficiency, stroke, intracranial bleeding) both preoperatively and intraoperatively [6,9]. This practice has been under intensive and emotional debate [11,12,13,14], since alpha-receptor blockade in particular may have unpleasant side effects for patients (e.g., orthostatic hypotension, reflex tachycardia) and may delay surgery due to drug titration over weeks in some patients. In addition, multiple analyses showed that alpha-receptor blockers (ARBs) cannot prevent intraoperative hypertensive episodes but rather may be associated with more hypotensive episodes postoperatively [15,16,17,18]. Whereas most centers still apply preoperative ARBs to all patients independent of individual symptoms or characteristics (as recommended in the guidelines [6,9]), other centers with a high case load avoid ARBs altogether, using other antihypertensive medications to control pre- and perioperative hypertension [12,15,16,19,20,21,22].

In the last three decades, transperitoneal or retroperitoneal endoscopic tumor resection has become the recommended and preferred technique for PPGL surgery [5]. The procedure is often associated with intraoperative episodes of catecholamine release and, subsequently, excessive hypertension and/or tachycardia. Hypotension, usually due to vasoplegia rather than hypovolemia [20,23], also occurs intraoperatively, independent of the surgical approach, but slightly more often in retroperitoneoscopic operations [24,25]. In the literature, hemodynamic fluctuations are often termed “hemodynamic instability” and must be expected in PPGL resection. Therefore, in experienced centers, anesthesiologists are prepared to counteract these sudden hemodynamic changes using vasoactive or anti-tachycardic drugs. Most often, sodium nitroprusside (SNP), urapidil, or short-acting calcium antagonists are given against hypertension, norepinephrine (NE) or phenylephrine (both alpha-adrenoreceptor agonists) against hypotension, and short-acting beta-blockers (esmolol) against tachycardia [15].

To date, multiple studies have analyzed the incidence and risk factors for excessive hemodynamic changes during endoscopic adrenalectomy using extremely inhomogeneous definitions of “hemodynamic instability” [24], with huge differences in cut-offs for both hypertensive and hypotensive events [15,18,24,26,27,28,29,30,31]. In addition, some authors also consider the application of vasoactive drugs as a “hemodynamic event” [24,29,30,32], whereas others do not [16,18,26,27,28], although drugs were administered as anesthesiologists’ reactions to prevent severe hemodynamic changes.

In various types of operations, even brief intraoperative episodes of hypotension have been documented to be relevant risk factors for negative postoperative outcomes, such as acute kidney injury, myocardial injury, and also 30-day mortality [33,34,35], and it is well accepted that hypotension should be avoided during the perioperative period [36]. To our knowledge, no analysis so far has focused on the incidence of and risk factors for intraoperative hypotension with or without hypertensive episodes during endoscopic surgery for PPGL, especially in the context of incidental and early diagnosis without typical symptoms of hormone excess. Therefore, we aimed to quantify these phenomena in a large consecutive series of patients with PPGL, identify predisposing factors, and discuss potential consequences for perioperative treatment.

## 2. Materials and Methods

### 2.1. Patients

**Figure 1:**
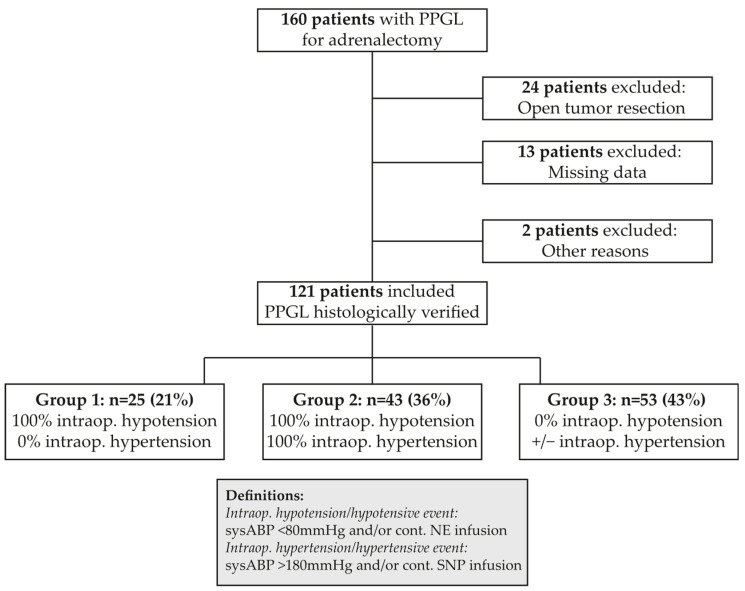
Study algorithm. PPGL: pheochromocytoma/paraganglioma; intraop.: intraoperative; sysABP: systolic arterial blood pressure; NE: norepinephrine; SNP: sodium nitroprusside.

Perioperative records of all patients with histologically confirmed PPGL treated at the Medical University of Vienna, Department of Anaesthesia, Intensive Care Medicine and Pain Medicine, Division of General Anaesthesia and Intensive Care Medicine, together with the Section of Endocrine Surgery, Division of General Surgery, Department of Surgery and the Clinical Division of Endocrinology and Metabolism, Department of Medicine, were collected prospectively and analyzed retrospectively. Of the 160 patients treated over a 2-decade period starting in 1998, 24 patients were not included in our analysis because the tumor resection was performed via open access. Another 15 patients were excluded due to missing perioperative data (n = 13), because surgery was not elective (n = 1; patient on ECMO due to acute catecholamine-induced heart failure), or PPGL resection was performed together with another intra-abdominal operation (n = 1); thus, 121 patients were included (excluding pregnant women, individuals <18 years) (see Figure 1).

### 2.2. Preoperative Diagnosis, Documentation, and Preoperative Treatment

Urinary catecholamines and/or metanephrines from a 24 h collection and/or plasma metanephrines were used to confirm the diagnosis of a catecholamine-producing tumor described on CT/MRI scans. Scans were performed either to search for an adrenal tumor because of the clinical suspicion of endocrine-related hypertension or during the workup of a hereditary disease (VHL, NF1, MEN2, and SDHx mutations). Moreover, in 49% of cases, tumors were diagnosed incidentally as an adrenal mass during CT/MRI scans for various other reasons (incidental diagnosis). After confirming the diagnosis biochemically, the patients were scheduled for surgery, and the type and duration of symptoms and medical history were documented. Three weeks before surgery, outpatient titration of ARBs was initiated using either phenoxybenzamine or doxazosin. Other antihypertensive drugs were reduced or stopped, targeting a blood pressure of <160/90 mmHg but >85/45 mmHg after rising upright quickly. Beta-blockers (Propanolol, Metoprolol, Bisoprolol) were used if necessary to lower the heart rate to <90/min and minimize extrasystoles in selected cases. Only 10 patients did not receive preoperative alpha-blockade owing to intolerance or organizational reasons.

### 2.3. Intraoperative Treatment

Endoscopic adrenalectomy was performed via the transperitoneal flank approach, resecting the adrenal en bloc with the tumor and surrounding fatty tissue. Tumor diameter was documented by the surgeon and pathologist; histology confirmed the diagnosis of PPGL in all patients.

The type of hypnotic (inhaled/total intravenous anesthesia [TIVA]) and opioid (fentanyl/remifentanil) used was determined by the anesthesiologist in charge. Epidural anesthesia was not used. Invasive arterial blood pressure (ABP) monitoring was performed, and a central venous catheter (CVC) was placed in all patients. SNP or nitroglycerin to treat hypertension and NE to treat hypotension were connected to a separate line of the CVC and used as a continuous infusion with the possibility of applying small bolus injections. Esmolol was administered as needed for the treatment of tachycardia or arrhythmias.

At our institution, during pheochromocytoma resection, systolic blood pressure >160 mmHg or <90 mmHg and heart rate >110/min are definitive indications for medical intervention. Crystalloid fluid was given at the discretion of the anesthesiologist.

### 2.4. Postoperative Surveillance

After surgery, patients were monitored continuously either in a post-anesthesia care unit (PACU) or, in selected cases, in an intensive care unit (ICU) until at least the next morning and remained in the hospital for an average of 7 days. Patients were routinely seen in the outpatient clinic 4–6 weeks after surgery.

### 2.5. Measurements

Patient characteristics, including medical history, type of diagnosis (incidentally during radiologic cross-sectioning imaging vs. specific symptoms for PPGL), patient size, height, tumor diameter, and maximal relative elevation of preoperative catecholamines (noradrenalin, adrenalin) or metanephrines (normetanephrines, metanephrines) were documented (hormones presented as fold of the upper normal limit [fold ULN]). In addition, continuous intraoperative data (application of medication, blood pressure, and heart rate) were recorded. For postoperative outcomes, the incidence of postoperative hypotension requiring continuous noradrenalin infusion in the PACU/ICU was documented together with the postoperative incidence of complications up to 1 month (myocardial infarction, arrhythmias, stroke, bleeding, and wound infection).

### 2.6. Definition of Hypotensive/Hypertensive Events

Both specific absolute blood pressure, thresholds, and the application of vasoactive medication were counted as hypo/hypertensive events, because vasoactive drugs were always administered in reaction to extreme hemodynamic changes (Figure 1).

“Hypotension” was defined as a minimum intraoperative systolic blood pressure of <80 mmHg, regardless of duration, as this is a generally accepted threshold for intraoperative hypotension that is associated with the threshold often used in the literature to indicate hypotension and is accepted as a definitive risk factor for adverse events [34,35]. Additionally, the continuous infusion of NE was considered a hypotensive event.

A rise in blood pressure >180 mmHg was used as the threshold for hypertension, as this is the definition of hypertensive emergency or grade 3 hypertension, and is also associated with an elevated risk of cardio- and cerebrovascular complications [37,38,39]. Equally, the application of SNP was counted as a hypertensive event.

### 2.7. Statistical Analysis

Data were analyzed using SPSS for Windows, version 28.0. As most parameters did not follow a normal distribution (Shapiro–Wilk test or visual inspection), non-parametric tests were used for the analysis of all parameters, and data are presented as median and interquartile range (IQR; 25th–75th percentile). For continuous parameters, the Kruskal–Wallis H-test was used, and for dichotomous parameters, Fisher’s exact test (Rx2) was used for group comparisons between patients within the three groups defined after the occurrence of intraoperative hypo- and/or hypertension. Group 1 included only patients with hypotension, group 2 included those with both hypotensive and hypertensive events, and group 3 included those without hypotension (Figure 1). Binomial logistic regression was used to analyze potential risk factors for hypotension without hypertension or hypotension. Baseline variables (age, sex, BMI) were combined with risk factors for perioperative hemodynamic instability documented in other analyses (tumor size [29,30,40], level of metanephrines/catecholamines [28,29,30,40], blood pressure before surgery [30], diabetes mellitus (DM) [28,41], age [28,41], alpha-blocker pretreatment [18,30], beta-blocker pretreatment [42]), together with factors not investigated before (incidental diagnosis, type of anesthesia, type of opioid used). After the univariate analysis, statistically significant factors were included in the multivariate model. For all tests, a *p*-value of <0.05 was considered statistically significant.

## 3. Results

The baseline information of all 121 patients included in the study is presented in Table 1. The median age at surgery was 51 (39–62) years. In total, 20.7% of tumors were based on germline mutations, and the vast majority were unilateral (92.6%) and benign (95.9%). Hypertension was present in 72.7% of patients preoperatively, whereas tachycardia (19.8%) and headache (17.4%) were rare.

Most patients received phenoxybenzamine as pretreatment before surgery; doxazosin was used in only 6.6% of patients, and 10 patients (8.3%) did not receive alpha-blockers at all. At the time of surgery, 28.9% of patients received concomitant beta-blocker therapy, and less than 20% still received other antihypertensive medications such as calcium-channel blockers or ACE inhibitors/AT2-receptor blockers.

Overall, by definition, at least one episode of intraoperative hypotension was documented in 68/121 (56.2%) cases. Thereof, 25/121 (20.7%) patients showed hypotension without hypertensive events during surgery.

**Figure 2:**
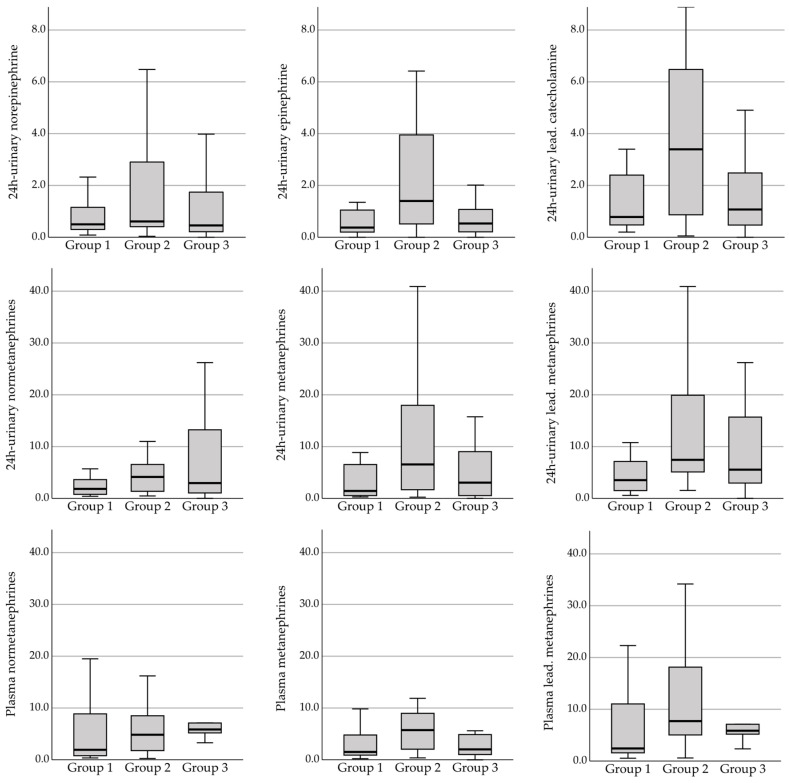
Catecholamine/metanephrine levels of the 3 groups (given as fold of upper limit normal [ULN]). Lead.: leading.

Table 2 presents the preoperative data comparing the three groups of patients: group 1 included patients with hypotension but without hypertensive events, group 2 included patients with hypotensive and hypertensive events, and group 3 included patients without hypotension. Comparing the three groups, there was a significant difference between all groups regarding the median age, pre-existing diabetes mellitus (DM), and type of tumor diagnosis (incidental finding vs. typical symptoms or genetic disease leading to testing for PPGL). Patients in group 1 and group 2 were older, had a higher proportion of DM, and were diagnosed incidentally more often. Tumor diameter did not show a significant difference among the three groups, whereas there was a clear trend toward the highest preoperative urinary and plasma catecholamine and metanepehrine levels documented in patients in group 2 (both intraoperative hypo- and hypertension; Table 2 and Figure 2). In particular, adrenalin and its metabolite metanephrine levels were higher in group 2 than in the other two groups. In contrast, patients showing only hypotensive events intraoperatively had the lowest median levels of both urine and plasma catecholamines and (nor-)metanephrines (see Table 2 and Figure 2). Group differences for urinary adrenalin, plasma metanephrines, and maximum leading urinary catecholamine and metanepehrine reached the level of significance. The applied phenoxybenzamine dose was not different between the groups, and the median systolic and diastolic blood pressure in all groups indicated that the target blood pressure was reached in the majority of patients. However, the preoperative ABP (under the final dose of antihypertensive medication) showed a clear trend to the lowest values in group 1 (systolic, *p* = 0.056; diastolic, *p* = 0.003).

In Table 3, the intraoperative and postoperative data are compared between the three groups. Group differences in the volume of intraoperative crystalloid infusion and proportion of patients receiving remifentanil intraoperatively showed statistically significant differences; in group 2 (hypotension + hypertension), patients received the highest amounts of intravenous fluids (median 3550 mL vs. 2500 mL in groups 1 + 3; *p* = 0.004), and the proportion of patients with intraoperative use of remifentanil was lowest in patients without hypotension (group 3).

According to the definition of the groups, there were significant differences in the dose of the maximum infusion rate of NE and SNP between the groups, and ABP also differed significantly. No difference was found in the maximum heart rate.

Postoperative data were available for 116 patients. Only patients who experienced intraoperative hypotension (n = 6/121; 5.0%; no patients in group 3) required postoperative noradrenalin infusion in the PACU/ICU due to prolonged hypotension. Three patients showed postoperative bleeding requiring reoperation, and four patients had minor local superficial wound infections (no significant group differences). No cardio- or cerebrovascular complications were documented.

Using univariate and multivariate binomial logistic regression analyses, risk factors and odds ratios for intraoperative hypotension without hypertension (group 1 vs. group 2 + 3; Table 4(a)) and hypotension in general (group 1 + group 2 vs. group 3, Table 4(b)) were analyzed. In univariate analysis, the significant risk factors for patients to show intraoperative hypotension without any hypertensive event were age (increase of 4.7% per year), incidental diagnosis (4.4-fold increase in risk when diagnosed incidentally), and preoperative diastolic ABP (7.2% decrease in risk per +1 mmHg). In the multivariate analysis, only age (4% risk increase) and preoperative diastolic ABP (risk decrease of 6% with higher diastolic ABP) remained significant; however, incidental diagnosis showed borderline significance (2.8-fold risk increase; *p* = 0.060).

Table 4(b) shows the results of the binomial logistic regression analysis for perceiving intraoperative hypotension with or without hypotension (group 1 + 2 vs. group 3). Age (risk increase of 3% per year) and incidental diagnosis (2.2-fold risk increase) reached significance in the univariate analysis. Additionally, the diagnosis of DM (3.7-fold risk increase with DM) and the use of remifentanil (3.2-fold risk increase with the use of remifentanil) were associated with the risk of intraoperative hypotension. In the multivariate analysis, only incidental diagnosis (risk increase 3.0-fold) and use of remifentanil (risk increase 3.8-fold) remained significant. However, DM showed borderline significance (3.2-fold increase in risk; *p* = 0.053).

## 4. Discussion

Despite preoperative treatment with ARBs, hemodynamic instability with hypertensive and hypotensive episodes remains a frequent phenomenon during PPGL resection. The frequency of these events is difficult to quantify because of the different definitions of hemodynamic instability in the literature. In most analyses, hypertensive episodes occur in 26 to 80% and intraoperative hypotension occurs in 25 to 80% [16,24,27,28,30,42,43]; however, detailed information concerning hypotension in particular is often difficult to extract from previous studies. This is mainly because of different cut-offs, the mixing up of hypertension/hypotension as “instability”, some not even counting the application of vasoactive drugs as an event, and the fact that many analyses focus on the observation or prevention of intraoperative hypertensive events alone. To date, only Takeda et al. [28] analyzed 68 patients (85% pretreated with doxazosin; median dose 3 mg) and provided detailed information on hypotension (defined as systolic ABP < 80 mmHg), differentiating between all hypotensive events (incidence 25.0%) and hypotension without concomitant hypertension (incidence 10.3%). Unfortunately, no information was provided in the study on how and when hypotension was treated and if anti-hypotensive treatment alone was also counted as an event [28].

In the current study, the overall incidence of intraoperative hypotension was 56.2% and 20.7% for hypotension without a concomitant hypertensive event. Generally, intraoperative hypotension during noncardiac surgery is a risk factor for perioperative complications such as kidney or myocardial injury, and there is a broad consensus that hypotension should be strictly avoided [36]. In particular, brief episodes of profound hypotension appear to be more dangerous than prolonged periods of only slightly decreased blood pressure [44], which is reflected by the cut-off for hypotension used in this analysis (systolic ABP < 80 mmHg).

The rationale for ARBs before PPGL surgery is to avoid pre- and intraoperative cardiovascular complications, especially to control high blood pressure peaks by blocking the overstimulation of alpha-adrenoreceptors [45]. However, multiple trials demonstrated that ARBs could not avoid intraoperative hemodynamic instability and were not superior to other antihypertensive pretreatment such as calcium-channel blockers [16,27,29,46]. On the contrary, ARBs were related to more and longer periods of peri- and postoperative hypotension or complications [30,42,47]. Guidelines still recommend ARB pretreatment [6,9] and they are also still included in our institutional standard. Consequently, 91.7% of the patients in this analysis received ARBs. However, the frequency of hypertensive events (61.2%) was only slightly higher than the frequency of hypotensive events (56.2%), and for the 20.7% of patients who never showed intraoperative hypertension but only hypotensive events, it seems difficult to find arguments for ARB premedication. Although it was not feasible to prove a causal relationship between ARBs and hypotension in our study, this medication could potentially complicate the treatment of hypotension by reducing the effectiveness of NE infusion to treat hypotension; NE acts as an agonist on alpha receptors, but these were blocked preoperatively [48].

To the best of our knowledge, this is the first study to evaluate the risk factors for intraoperative hypotension without hypertension. In the univariate analysis, age, incidental diagnosis, and lower preoperative diastolic ABP were risk factors. In the multivariate analysis, age and diastolic ABP still reached a level of significance, whereas incidental diagnosis showed only borderline significance. Age as a risk factor seems obvious, as intraoperative hypotension is frequent in older patients [49], and has also been shown to be a perioperative risk factor during PPGL surgery in other studies [28,41]. The median age in the group “hypotension only” was 60 years, which was higher than the median age in most other analyses (usually 50–55 years [18,27,28,50]) and clearly higher compared to patients without hypotension in the analyzed cohort. The calculated risk increase for intraoperative hypotension in this study was 40% for every decade. An additional argument against using ARB in PPGL surgery routinely and in general against hypertension is the occurrence of more side effects in elderly patients, especially orthostatic hypotension [51].

The higher rate of incidentally diagnosed PPGL in patients with hypotension reflects the fact that an increasing number of patients are diagnosed without typical symptoms and at higher ages in Western countries [3,4,52]. Although a high proportion of these patients have arterial hypertension, it is most often essential and not hormonally triggered [8]. In total, 19 of the 25 (76%) patients with hypotension were diagnosed without classical symptoms (such as hypertensive attacks, tachycardia, and diaphoresis) but incidentally during various radiological imaging. Shao et al. [47] and Issacs et al. [53] already questioned the necessity of alpha-receptor blockade in “atypical” PPGL without symptoms as for these patients, the risk of prolonged hypotension and the need for vasoactive treatment were increased. Issacs et al. [53] recommended individual and interdisciplinary decision on preoperative treatment, also considering the secretory profile of the endocrine tumor. Although the level of catecholamines/metanephrines could not be evaluated as risk factors for intraoperative hypotension in our analysis, the median levels of all catecholamines/metanephrines were lower in patients with “hypotension only” (see Figure 2). In fact, in the “hypotension only” group, in 57% of patients, the leading plasma metanephrine level was lower than 5-fold of the ULN; in contrast, 80% of patients with intraoperative hypertensive events showed an increase in the leading plasma metanephrines of more than 5-fold of the ULN. Although absolute prediction of both hypotension and hypertension was impossible based on the level of catecholamines/metanephrines in any study to date, many analyses have shown a correlation between the secretion profile and hemodynamic variability [28,29,30,40]. Preoperatively, both diastolic (significant) but also systolic (borderline-significant) ABP were lower in the “hypotension only” group. Especially the lower diastolic ABP might also reflect the lower catecholamine secretion of these tumors in this group resulting in lower alpha-receptor-mediated vasoconstriction.

The 43 patients (35.5%) with a combination of intraoperative “hypotension and hypertension” (group 2) showed different baseline characteristics. They were younger than patients with “hypotension only” (group 2 vs. group 1: 51 vs. 60 years), and still older than those “without hypotension” (group 3; median 46 years). Additionally, this group showed the highest catecholamine levels, particularly the highest levels of epinephrine and its metabolites. The median intraoperative maximum systolic ABP was highest and the median minimum systolic ABP was lowest in this group, so this group showed “classical hemodynamic instability”. This group also had the highest proportion of patients with DM, most likely due to high epinephrine activity impairing glucose metabolism [54,55,56,57]. High catecholamine secretion might be the cause of hemodynamic instability [28,29,30,40]; however, DM itself was also identified as a risk factor by Takeda et al. [28]. A potential explanation could be autonomic cardiovascular neuropathy known to be associated with intraoperative hemodynamic instability in general [58].

In the univariate analysis, significant risk factors for hypotension in general were age, incidental diagnosis, DM, and interestingly, the intraoperative use of remifentanil. In the multivariate analysis, only incidental diagnosis (OR: 3.0) and remifentanil (OR 3.7) were significant, whereas DM showed borderline significance (*p* = 0.053; OR 3.12). The fact that “incidental diagnosis” is also a relevant factor when analyzing the two heterogenic groups of hypotensive patients together might indicate that a lot of patients even with tumors with a high potential to secrete catecholamines are “asymptomatic”, e.g., because of the down-regulation of adrenoreceptors [59] or symptoms not being recognized correctly [60].

To date, remifentanil has not been identified as a risk factor for intraoperative hypotension in PPGL resection. Although remifentanil might have the potential to suppress catecholamine release during the creation of pneumoperitoneum and therefore might be an interesting option for laparoscopic PPGL surgery [61], it has also been associated with a higher incidence of intraoperative hypotension and bradycardia in various other surgical indications [62] and can even be used to actively reduce blood pressure when used at higher doses [63,64]. Therefore, it should be used carefully during endoscopic adrenalectomy, especially when hormone levels decrease to normal after tumor resection and systemic vascular resistance drops [23,65].

As age, lower preoperative blood pressure, incidental diagnosis, DM, and higher metanephrine levels are potential risk factors for intraoperative hypotension in PPGL surgery, ARBs, which are also known to be associated with intraoperative hypotension, should be used with caution. Some centers have already recommended avoiding ARBs at all and using other antihypertensive drugs to normalize blood pressure preoperatively [14,15,47] or individualize ARB therapy based on preoperative symptoms [66]. Others have proposed using reduced doses of ARBs rather than escalating the dose to have both the theoretical protective effects of ARBs without their hypotensive potential [18,53]. This seems to be a plausible approach, especially for patients who are at risk of intraoperative hypertension. However, escalating ARBs to the high doses recommended historically or in guidelines leads to a very high frequency of perioperative hypotension: in the PRESCRIPT trial [50], designed to compare the effect of pretreatment with phenoxybenzamine or doxazosin, patients received a median dose of 120 mg/day phenoxybenzamine and 40 mg/day of doxazosin (our cohort: median dose phenoxybenzamine: 45 mg; doxazosin 6 mg). Hypotension occurred in 74.2–80.9% of patients, and 37.8–41.2% of patients needed two or more vasoactive/inotropic drugs intraoperatively. Moreover, 32.4–33.3% needed postoperative vasopressors (our cohort: 5.2%).

The limitations of this study are its retrospective design and the fact that hypotensive episodes were only documented absolutely but not quantitatively or qualitatively. However, as any hypotensive event should be avoided whenever possible, it is important to indicate patients at highest risk for hypotension, and this could be achieved.

As a strength, we conducted a detailed analysis of intraoperative hypotension and its risk factors in a large cohort of patients with PPGL treated in a single center with a uniform pre- and intraoperative protocol.

## 5. Conclusions

Higher age, incidental diagnosis, lower diastolic ABP, and DM were preoperative risk factors for intraoperative hypotension.

In older patients and in diabetic patients with PPGL diagnosed incidentally during radiologic imaging and with low plasma metanephrines (maximum levels, e.g., lower 5-fold of ULN) in particular, ARB should be used with caution or even avoided to prevent hypotension. Additionally, remifentanil should be used carefully so as not to promote hypotension, and other intraoperative opioids should be considered for patients at risk.

The findings underscore the need for a multidisciplinary team approach (anesthesiologist, surgeon, endocrinologist) to perioperative management, including the use of ARBs and intraoperative opioids, to ensure personalized care for patients based on their individual risk factors.

## Figures and Tables

**Table 1:** Baseline data, all patients.

		n	%
		121	100
**Sex**			
	Female	63	52.1
	Male	58	47.9
**Tumor type**			
	Pheochromocytoma	117	96.7
	Paraganglioma	4	3.3
**Genetic disease**			
	Yes	25	20.7
	NF1	5	4.1
	MEN2A	14	11.6
	MEN2B	1	0.8
	VHL	5	4.1
	No	96	79.3
**Side**			
	Unilateral	112	92.6
	Bilateral	9	7.4
**Dignity**			
	Benign	116	95.9
	Malignant	5	4.1
**Arterial hypertension**			
	Yes	88	72.7
	No	33	27.3
**Tachycardia**			
	Yes	24	19.8
	No	97	80.2
**Headache**			
	Yes	21	17.4
	No	100	82.6
**Coronary heart disease**			
	Yes	12	9.9
	No	109	90.1
**Cerebrovascular disease**			
	Yes	7	5.8
	No	114	94.2
**Diabetes mellitus**			
	Yes	24	19.8
	No	97	80.2
**Alpha-Adreno receptor blocker**			
	Phenoxybenzamine	103	85.1
	Doxazosin	8	6.6
	None	10	8.3
**Other antihypertensive medication**			
	Beta-blocker	35	28.9
	ACE inhibitor/AT2 blocker	24	19.8
	Calcium-channel blocker	21	17.4

NF1: Neurofibromatosis Type 1; MEN2A: Multiple Endocrine Neoplasia 2A; MEN2B: Multiple Endocrine Neoplasia 2B; VHL: Von Hippel–Lindau; ACE: angiotensin-converting enzyme; AT2: angiotensin-2 receptor.

**Table 2:** Preoperative data: groups divided by intraoperative hypotension +/− hypertension.

		Group 1	Group 2	Group 3	*p*
		Hypotension Only	Hypotension + Hypertension	No Hypotension	
n		25	43	53	
**Age—median, years**					
		60 (47–68)	51 (38–64)	46 (37–55)	0.011
**Sex—n**					
	female	17	18	28	0.116
	male	8	25	25	
**BMI—median, kg/m^2^**					
		25.4 (22.6–29.8)	24.5 (22.2–27.7)	24.8 (21.9–28.1)	0.585
**Diabetes mellitus—n**					
	Yes	6	13	5	*0.028*
	No	19	30	48	
**Incidental diagnosis—n**					
	Yes	19	20	20	*0.006*
	No	6	23	33	
**Tumor diameter—median, mm**					
		38 (30–45)	40 (30–50)	40 (28–50)	0.508
24 h urinary noradrenalin—median, fold ULN *					
		0.5 (0.3–1.2)	0.6 (0.4–2.9)	0.5 (0.2–1.7)	0.282
24 h urinary adrenalin—median, fold ULN *					
		0.4 (0.2–1.1)	1.4 (0.5–4.0)	0.5 (0.2–1.1)	*0.004*
24 h urinary leading catecholamine—median, fold ULN *					
		0.8 (0.5–2.4)	3.4 (0.9–6.5)	1.1 (0.5–2.5)	*0.002*
**24 h urinary normetanephrine—median, fold ULN ^+^**					
		1.8 (0.7–3.7)	4.1 (1.4–6.5)	3.0 (1.0–13.2)	0.345
**24 h urinary metanephrine—median, fold ULN ^+^**					
		1.4 (0.5–6.9)	6.6 (1.7–180.0)	3.0 (0.5–9.0)	0.063
**24 h urinary leading metanephrine—median, fold ULN ^+^**					
		3.5 (1.4–7.3)	7.4 (5.1–19.9)	5.5 (3.0–15.7)	*0.038*
**Plasma normetanephrine—median, fold ULN ^#^**					
		1.9 (0.8–8.9)	4.8 (1.8–8.5)	5.9 (5.2–7.1)	0.225
**Plasma metanephrine—median, fold ULN ^#^**					
		1.5 (0.9–4.8)	5.7 (2.1–9.0)	2.0 (1.0–4.9)	*0.019*
**Plasma leading metanephrine—median, fold ULN ^#^**					
		2.4 (1.6–11.0)	7.7 (5.0–18.1)	5.9 (5.2–7.1)	0.065
**Dose phenoxybenzamine—median, mg**					
		45 (20–60)	48 (25–60)	50 (30–75)	0.623
**Preoperative ABP systolic—median, mmHg**					
		133 (125–146)	147 (132–153)	140 (130–150)	0.056
**Preoperative ABP diastolic—median, mmHg**					
		77 (70–80)	83 (80–90)	82 (79–90)	*0.003*
**Preoperative heart rate—median, bpm**					
		82 (70–91)	88 (75–98)	82 (70–90)	0.175

Information given as absolute numbers (n) or median (25th–75th percentile); * 24 h urinary catecholamines: 103 patients; ^+^ 24 h urinary metanephrines: 57 patients; ^#^ plasma metanephrines: 70 patients; BMI: body mass index; fold ULN: fold upper limit normal; ABP: arterial blood pressure; bpm: beats per minute.

**Table 3:** Intraoperative and postoperative data: groups divided by intraoperative hypotension +/− hypertension.

		Group 1:	Group 2:	Group 3	*p*
		Hypotension Only	Hypotension + Hypertension	No Hypotension	
n		25	43	53	
**Patients with intraoperative hypotensive event(s)**					
		25 (100%)	43/43 (100%)	0/53 (0%)	
**Patients with intraoperative hypertensive event(s)**					
		0/25 (0%)	43/43 (100%)	31/53 (58.5%)	
**Duration of surgery—median, min**					
		105 (90–148)	120 (98–155)	103 (80–135)	0.237
**Intraoperative crystalloid infusion—median, ml**					
		2500 (1500–3750)	3550 (2500–4500)	2500 (2000–3500)	*0.004*
**Type of anesthesia—n**					
	Inhalative	23	37	47	0.816
	TIVA	2	6	6	
**Remifentanil used—n**					
	Yes	13	25	15	*0.008*
	No	12	17	37	
**Intraoperative beta-blocker administration—n**					
	Yes	7	16	12	0.303
	No	18	27	41	
**Maximum intraoperative noradrenalin infusion rate—median, µg/kg/min**					
		0.05 (0.00–0.10)	0.04 (0.00–0.18)	0.00 (0.00–0.00)	*<0.001*
**Maximum intraoperative sodium-nitroprusside infusion rate—median, µg/kg/min**					
		0.00 (0.00–0.00)	0.80 (0.10–1.60)	0.00 (0.00–1.40)	*<0.001*
**Maximum intraoperative ABP systolic—median, mmHg**					
		134 (128–151)	185 (160–211)	160 (138–190)	*<0.001*
**Maximum intraoperative ABP diastolic—median, mmHg**					
		70 (64–79)	90 (77–104)	85 (77–97)	*<0.001*
**Minimal intraoperative ABP systolic—median, mmHg**					
		78 (74–87)	75 (66–83)	95 (88–101)	*<0.001*
**Minimal intraoperative ABP diastolic—media, mmHg**					
		48 (40–49)	41 (39–46)	55 (49–60)	*<0.001*
**Maximum intraoperative heart rate—median, bpm**					
		91 (81–111)	102 (90–120)	100 (84–116)	0.436
Postoperative noradrenalin infusion (PACU, ICU)—n *					
	Yes	2	4	0	*0.049*
	No	22	38	50	
Postoperative bleeding—n *					
	Yes	0	2	1	0.596
	No	24	41	51	
Postoperative infection—n *					
	Yes	0	3	1	0.415
	No	24	40	51	

Information given as absolute numbers (n) or median (25th–75th percentile). * information available for 116 patients. TIVA: total intravenous anesthesia; ABP: arterial blood pressure; PACU: post-anesthesia care unit; ICU: intensive care unit; bpm: beats per minute.

**Table 4:** (**a**) Univariate and multivariate binominal logistic regression analysis for selected risk factors for intraoperative hypotension. Patients with hypotension only (group 1; n = 25) compared to all other patients (group 2 + 3; n = 96). (**b**) Univariate and multivariate binominal logistic regression analysis for selected risk factors for intraoperative hypotension. All patients with hypotension (group 1 + 2; n = 68) compared to those without hypotension (group 3; n = 53).

(**a**)				
**Risk Factor**	**Crude OR**	***p***	**Adjusted OR**	***p***
Sex	0.433	0.078		
Age	1.047	0.008	1.039	0.034
BMI	1.051	0.216		
Incidental diagnosis	4.433	0.004	2.844	0.060
Diabetes mellitus	1.368	0.559		
Tumor diameter	0.996	0.786		
24 h urinary noradrenalin	0.785	0.137		
24 h urinary adrenalin	0.861	0.207		
24 h urinary max. leading catecholamine	0.808	0.059		
24 h urinary normetanephrine	0.969	0.523		
24 h urinary metanephrine	0.970	0.303		
24 h urinary max. leading metanephrine	0.961	0.196		
Plasma normetanephrine	0.966	0.319		
Plasma metanephrine	0.933	0.155		
Plasma max. leading metanephrine	0.953	0.150		
Preoperative beta-blocker	1.203	0.704		
Dose phenoxybenzamine	0.993	0.407		
Preoperative ABP systolic	0.981	0.178		
Preoperative ABP diastolic	0.928	0.003	0.939	0.015
Type of anesthesia (reference TIVA)	1.643	0.534		
Use of remifentanil (reference no remifentanil used)	1.432	0.400		
(**b**)				
**Risk Factor**	**Crude OR**	***p***	**Adjusted OR**	***p***
Sex	1.056	0.882		
Age	1.030	0.025	1.019	0.190
BMI	0.997	0.929		
Incidental diagnosis	2.219	0.033	3.026	0.011
Diabetes mellitus	3.722	0.015	3.156	0.053
Tumor diameter	1.009	0.454		
24 h urinary noradrenalin	1.003	0.970		
24 h urinary adrenalin	1.184	0.071		
24 h urinary max. leading catecholamine	1.100	0.108		
24 h urinary normetanephrine	0.950	0.251		
24 h urinary metanephrine	1.028	0.371		
24 h urinary max. leading metanephrine	1.011	0.684		
Plasma normetanephrine	0.999	0.983		
Plasma metanephrine	1.027	0.369		
Plasma max. leading metanephrine	1.017	0.450		
Preoperative beta-blocker	1.056	0.894		
Dose phenoxybenzamine	0.993	0.289		
Preoperative ABP systolic	1.010	0.360		
Preoperative ABP diastolic	0.980	0.229		
Type of anesthesia (reference TIVA)	0.940	0.957		
Use of remifentanil (reference no remifentanil used)	3.232	0.003	3.752	0.002

OR: odds ratio; BMI: body mass index; ABP: arterial blood pressure; TIVA: total intravenous anesthesia.

## Data Availability

Data are unavailable due to ethical restrictions.
